# Cut off values of waist circumference & associated cardiovascular risk in egyptians

**DOI:** 10.1186/1471-2261-11-53

**Published:** 2011-08-16

**Authors:** M Mohsen Ibrahim, Ahmed A Elamragy, Hanan Girgis, Mona A Nour

**Affiliations:** 1 Cardiology Department - Cairo University Faculty of Medicine, Kasr Al-Aini St., Cairo, 11562, Egypt; 2 Information and Decision Support Center (IDSC), 1 Magless El Shaàb st., Cairo, 11582, Egypt; 3 Tropical Medicine Department - Cairo University Faculty of Medicine, Kasr Al-Aini St., Cairo, 11562, Egypt

**Keywords:** Abdominal Obesity, Waist Circumference, Risk Factors, Developing Countries, Egypt

## Abstract

**Background:**

Recent guidelines stressed the need to adopt different values of waist circumference (WC) measurements to define abdominal obesity in different ethnic groups. The aim of this study is to identify WC cutoff points in normotensive and hypertensive subjects which are diagnostic of abdominal obesity in a Middle Eastern population and the prevalence of abdominal obesity in a nationwide sample.

**Methods:**

Data were collected during phase-2 of the Egyptians National Hypertension Project survey. Blood pressure, anthropometric measurements and laboratory studies were performed according to a standardized protocol by trained personnel. To derive the cutoff points for WC, we applied the factor analysis on CV risk factors: diabetes mellitus, decrease in HDL-C and increase in LDL-C, triglycerides and left ventricular mass index by echocardiography.

**Results:**

The sample included 2313 individuals above the age of 25 years. WC values (mean ± SD) were 88 ± 14 cm and 95 ± 14 cm for normotensive (NT) and hypertensive (HT) men respectively, and 89.6 ± 14.7 cm and 95.7 ± 15.9 cm for NT and HT women respectively. Applying factor analysis, the weighted average cutoff points were 93.5 cm for both NT and HT men and 91.5 and 92.5 cm for NT and HT women respectively. Based on these thresholds, the prevalence of abdominal obesity was 48% in men and 51.5% in women.

**Conclusion:**

This is the first report of specific abdominal obesity cutoff points in a Middle Eastern country. The cutoff points were different from the Europid standards. There is a high prevalence rate of abdominal obesity among Egyptians which is associated with increased prevalence of cardiometabolic risk factors.

## Background

Abdominal obesity has recently been considered an established cardiometabolic risk factor (1,2). Individuals with abdominal obesity are at a great risk of developing diabetes and atherosclerotic cardiovascular disease (CVD) (1-4). It comes next to elevated plasma lipids as a population-attributable risk factor for acute myocardial infarction (5). Abdominal obesity was considered by the IDF as a prerequisite for the diagnosis of the metabolic syndrome (3).

The diagnosis of abdominal obesity in routine clinical practice depends on the measurement of waist circumference (WC). In Egypt as well as other Arab and Middle Eastern countries, the thresholds of WC diagnostic of abdominal obesity are arbitrary and were derived from European data. Gender-specific cutoff points were originally developed from a regression curve that identified the WC values associated with body mass index (BMI) ≥ 30 kg/m^2 ^in a large heterogenous sample of white men and women(1) and are not based on association with CVD risk factors. WHO and IDF recommended that the WC cutoff points used to define central (abdominal) obesity should be different among different ethnic groups(3,6) and that the Europid standards should be used in our Eastern Mediterranean region until national data become available. Identification of the normal cutoff values for each population is needed for health policy planners when developing CVD prevention programs, since universal criteria do not apply on all races.

In this study, we took advantage of the data collected from the Egyptian National Hypertension Project (NHP) survey to identify the cutoff points of WC diagnostic of abdominal obesity in Egyptian men and women and to examine the prevalence of abdominal obesity and its related cardiometabolic risk factors (RFs) among Egyptians. We used the principal component factor analysis (7,8) - which incorporates multiple RFs into a single variable - to derive appropriate WC cutoff points.

## Methods

### Study population

Data of WC in Egyptians were collected during phase-2 of the Egyptian NHP survey. The survey was conducted in 21 sampling locations in six Egyptian governorates, representing all Egypt's geographic areas and socioeconomic groups. The details of sample design, field operations, and technique for blood pressure measurements, laboratory tests and echocardiographic examination were reported in previous publications (9-11).

The sample could not be considered representative of the Egyptian population as in the second phase of the survey, the hypertensive individuals were over-sampled. Data of normotensives (NT) and hypertensives (HT) were analyzed separately.

WC measurements were taken using an anthropometric centimeter-measuring tape. WC was measured from the horizontal plane midway between the lowest lateral border of the rib cage and iliac crest.

Data about the socioeconomic status (SES) and urban/rural residence were derived from the questionnaire in phase 1 of the survey. Participants were classified into three groups based on the SES: high, mid and low (8). All participants gave their consent to participate in the survey. The protocol was approved by the Institution Review Board.

### Definition of Risk Factors

We used the following criteria for defining the RFs: elevated LDL-cholesterol; ≥130 mg/dL, low HDL-cholesterol; < 50 mg/dL in women and 40 mg/dL in men, increased triglycerides (TG); ≥ 150 mg/dL. Diabetes mellitus was defined as fasting blood glucose ≥ 126 mg/dL and/or post-prandial blood glucose ≥ 200 mg/dL. Left ventricular hypertrophy (LVH) was diagnosed if left ventricular mass index (LVMI) was > 125 gm/m^2 ^in men and > 110 gm/m^2 ^in women (12).

### Statistical Analysis

The statistical analysis was done using SPSS version 16. Continuous variables were expressed as gender-specific means and standard deviations while discrete variables were expressed as gender-specific proportions. All statistics were done for NT normotensive and HT hypertensive men and women separately. WC was divided into 6 categories (< 75, 75-, 85-, 95-, 105 and > 114.9 cm). The RFs data was tested against normality assumption. Tests showed that the data was not normally distributed. So, non-parametric tests were used to test the differences among different categories. We compared the differences in RFs within different WC categories using Kruskal-Wallis test for the numerical variables and Chi square test for the categorical variables. Differences were considered statistically significant at p < 0.05.

The principal component factor analysis (7, 8) was used in this study to identify the cutoff points diagnostic of abdominal obesity. WC cutoff points were based on the association with cardiometabolic RFs. Five risk factors were used: low HDL-C, high LDL-C, high TG, diabetes and LVH. The five RFs were used in a factor analysis model to obtain a factor (first factor) that accounts for most of the variability in the data, and was used to determine the cutoff point of the WC through the logistic regression technique.

The obtained factor was used as the outcome variable with 2 categories (< median and ≥ median), and the WC as the independent variable. Logistic regression predicted probabilities were used to construct the Receiver Operating Characteristic (ROC) curves for men and women separately. ROC curves were also estimated for NT and HT individuals separately to avoid the effect of oversampling HT individuals. The development of ROC curves allowed the identification of the optimal cutoff point which was considered in this study to be the point that maximizes both specificity and sensitivity. This occurs when specificity and sensitivity become almost equal.

## Results

### Baseline characteristics of the study subjects

The basic characteristics of the study subjects and the prevalence of risk factors are shown in table (1). The sample included 2313 individuals, 981 men and 1332 women. There was 754 NT) and 1559 (HT) individuals. Data of WC were available for 964 men and equal number of women. The age ranged between 25 and 90 years. The majority lived in urban areas (73.3%), while 26.7% lived in rural areas.

### WC Measurements

For NT and HT men, the mean WC values were 88 ± 14 and 95 ± 14 cm respectively while for NT and HT women, they were 89.6 ± 14.7 and 95.7 ± 15.9 cm respectively. (Table 1)

**Table 1:** Basic characteristics of the study subjects

	NT men (n = 311)	HT men (n = 670)	NT women (n = 443)	HT women (n = 889)
Age (years)^a^	46.5 ± 13.7	55.1 ± 13.1	42 ± 11.4	53.9 ± 11.6
WC (cm)^a^	88 ± 14.2	95 ± 14.4	89.6 ± 14.7	95.7 ± 15.9
BMI (kg/m^2^)^a^	25.9 ± 5	28.4 ± 6.1	29.2 ± 6.7	31.7 ± 7.5
SES (%)				
Low	41.8	36.4	45.8	44.8
Mid	49.5	52.3	46	46.2
High	8.7	11.3	8.1	9
Geographic region (%)				
Urban	71.1	73.6	70.4	73.1
Rural	28.9	26.4	29.6	26.9
Risk factors (%)				
DM	7.7	26.9	8.5	24.2
LVH	5.9	19.2	3.1	16.2
Decreased HDL-cholesterol	51.2	52.1	70.6	69.8
Elevated LDL-cholesterol	28.5	36.1	35.8	47.3
Elevated TG	29.3	46.1	24.8	39

^a ^The values are represented as means ± SD

Metabolic risk factors were defined according to the IDF criteria (9). Elevated TG ≥ 150 mg/dL; reduced HDL-cholesterol < 40 mg/dL for men and < 50 mg/dL for women; elevated LDL-cholesterol ≥ 130 mg/dL; DM when fasting blood glucose ≥ 126 mg/dL or postprandial blood glucose ≥ 200 mg/dL; LVH when LVMI > 125 gm/m^2 ^in men and > 110 gm/m^2 ^in women.

### Risk factors according to WC category

Prevalence of RFs in different WC categories is shown in table 2. Increased WC was associated with a higher prevalence rate for all RFs with the exception of LVH in men and HT women. The levels of plasma lipids, glucose and LVMI were linearly related to WC measurements in both NT and HT men and women (table 3) except for HDL in NT women and LVMI in HT men and women.

**Table 2:** Prevalence of risk factors (%) in different WC categories among NT and HT men and women

Risk Factors	WC category (cm)					
	< 75	75-	85-	95-	105-	> 114.9
**NT men**						
DM	2.1	2.5	6.8	7.5	23.1	41.7
LVH	4.3	9.0	6.6	3.8	0	0
High TG	4.3	17.3	34.2	48.1	42.3	75.0
Low HDL	44.7	45.7	50.0	58.5	61.5	58.3
High LDL	17.0	21.0	29.6	46.2	19.2	50.0
**HT men**						
DM	6.9	11.9	14.0	20.7	21.8	34.7
LVH	30.6	24.3	15.8	15.0	21.0	24.3
High TG	12.1	24.7	48.0	52.3	62.7	63.3
Low HDL	34.5	42.9	52.6	56.9	58.2	51.0
High LDL	22.4	27.4	35.1	36.7	43.9	49.0
**NT women**						
DM	4.4	4.3	8.9	12.0	8.8	20.0
LVH	2.9	1.1	0.9	3.4	6.3	18.2
High TG	8.8	16.1	25.9	29.3	47.1	52.0
Low HDL	66.2	67.7	70.5	71.7	85.3	72.0
High LDL	22.1	31.2	34.2	40.4	52.9	45.8
**HT women**						
DM	16.0	16.0	17.9	27.2	32.6	32.7
LVH	20.8	19.2	11.1	16.0	15.0	21.3
High TG	9.3	27.5	37.3	46.0	47.2	50.5
Low HDL	54.7	63.4	71.1	70.3	75.0	77.8
High LDL	40.5	38.5	38.7	56.0	49.7	58.6

**Table 3:** Linear trends in plasma lipid and glucose levels (mg/dl) and LVMI (gm/m^2^) in relation to different WC groups among NT and HT men and women

Risk Factors	WC category (cm)						Linearity
	< 75	75-	85-	95-	105-	> 114.9	p
**NT men**							
HDL	42 ± 10	41 ± 9	41 ± 10	38 ± 9	37 ± 7	40 ± 14	0.013
LDL	107 ± 24	105 ± 32	115 ± 32	128 ± 35	111 ± 27	152 ± 73	< 0.001
TG	106 ± 30	110 ± 41	154 ± 92	155 ± 66	172 ± 86	200 ± 71	< 0.001
FBS	86 ± 13	88 ± 16	92 ± 25	97 ± 42	112 ± 54	119 ± 35	< 0.001
PP	104 ± 28	111 ± 42	120 ± 46	117 ± 63	140 ± 72	140 ± 47	0.003
LVMI	89.7 ± 21	89.4 ± 25	89.3 ± 23	87.5 ± 21	89.4 ± 16	93.9 ± 16	0.997
**HT men**							
HDL	44 ± 9	42 ± 10	40 ± 10	39 ± 10	39 ± 10	40 ± 11	0.021
LDL	108 ± 39	110 ± 33	118 ± 37	123 ± 42	129 ± 43	124 ± 35	0.003
TG	102 ± 38	132 ± 62	164 ± 88	177 ± 104	181 ± 84	194 ± 103	< 0.001
FBS	90 ± 16	108 ± 71	102 ± 42	105 ± 48	112 ± 52	123 ± 68	0.018
PPBs	111 ± 32	128 ± 64	135 ± 70	145 ± 73	146 ± 73	167 ± 69	0.005
LVMI	109.3 ± 35	101.6 ± 33	100.4 ± 29	99.95 ± 29	102.8 ± 32	114.2 ± 54	0.727
**NT women**							
HDL	45 ± 11	44 ± 10	44 ± 11	44 ± 10	40 ± 9	45 ± 11	0.152
LDL	108 ± 28	115 ± 43	119 ± 37	120 ± 35	134 ± 38	123 ± 36	0.002
TG	99 ± 40	114 ± 48	124 ± 57	148 ± 98	142 ± 64	173 ± 90	< 0.001
FBS	95 ± 47	93 ± 22	96 ± 39	104 ± 98	97 ± 34	114 ± 52	0.032
PPBs	116 ± 51	118 ± 42	116 ± 41	131 ± 61	113 ± 34	147 ± 89	0.043
LVMI	73.7 ± 16	76.3 ± 18	75.7 ± 16	77.8 ± 16	85.1 ± 17	90.4 ± 25	< 0.001
**HT women**							
HDL	48 ± 12	46 ± 11	45 ± 10	44 ± 10	43 ± 10	42 ± 11	< 0.001
LDL	120 ± 38	122 ± 36	123 ± 39	139 ± 45	137 ± 45	148 ± 50	< 0.001
TG	106 ± 44	137 ± 71	148 ± 67	161 ± 81	160 ± 75	168 ± 76	< 0.001
FBS	96 ± 21	102 ± 31	106 ± 46	122 ± 69	123 ± 63	127 ± 63	< 0.001
PPBs	135 ± 53	131 ± 47	147 ± 72	159 ± 88	159 ± 79	167 ± 82	< 0.001
LVMI	94.5 ± 38	92.6 ± 32	89.4 ± 24	93.3 ± 25	92.4 ± 25	95.2 ± 22	0.638

HDL: High density lipoprotein cholesterol

LDL: Low density lipoprotein cholesterol

TC: Total cholesterol

FBs: fasting blood sugar

PPBs: post prandial blood sugar.

### WC Cutoff Points for Abdominal Obesity

**Figure 1:**
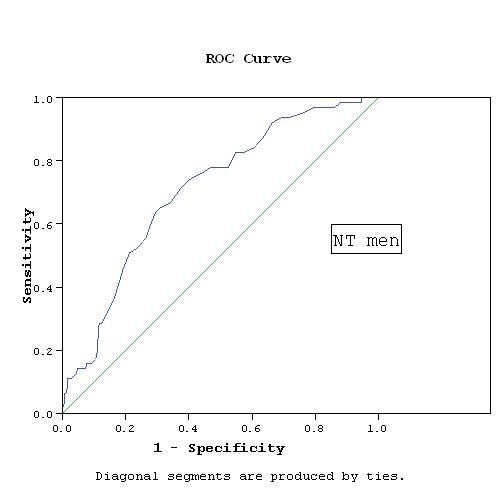
**WC cutoff point by ROC curve in NT men**. AUC = 0.78, p < 0.001 CI: 0.685- 0.876.

**Figure 2:**
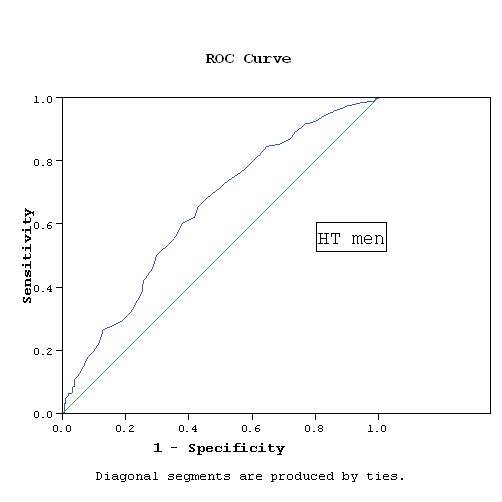
**WC cutoff point by ROC curve in HT men**. AUC = 0.668, p < 0.001 CI: 0.618- 0.718.

**Figure 3:**
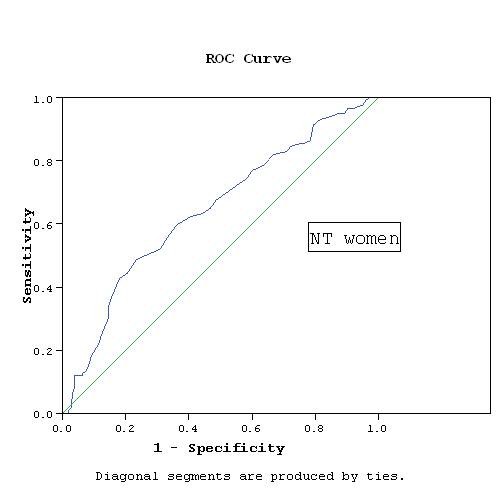
**WC cutoff point by ROC curve in NT women**. AUC = 0.697, p < 0.001 CI: 0.619- 0.776.

**Figure 4:**
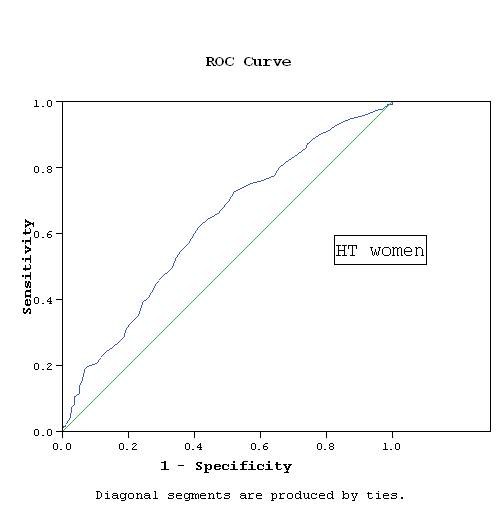
**WC cutoff point by ROC curve in HT women**. AUC = 0.63, p < 0.001 CI: 0.587- 0.673.

WC cutoff points were determined based on the association with RFs. Applying the score of the factor analysis and determining the cutoff points using the ROC curves, the cutoff points were 93.5 cm for NT and HT men, while they were 91.5 cm for NT women and 92.5 cm for HT women. The area under ROC curve was 0.78 and 0.67 in NT and HT men respectively, while it was 0.7 and 0.63 in NT and HT women respectively (Figures 1, 2, 3, 4).

### Prevalence of Abdominal Obesity

**Figure 5:**
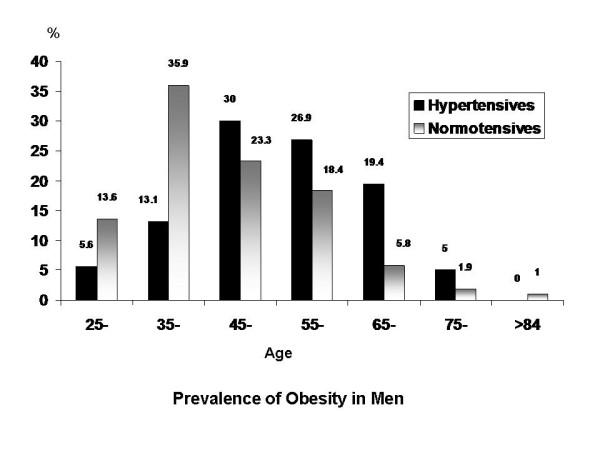
**Prevalence of Abdominal Obesity(%) in Different Age Decades in Normotensive and Hypertensive Men**.

**Figure 6:**
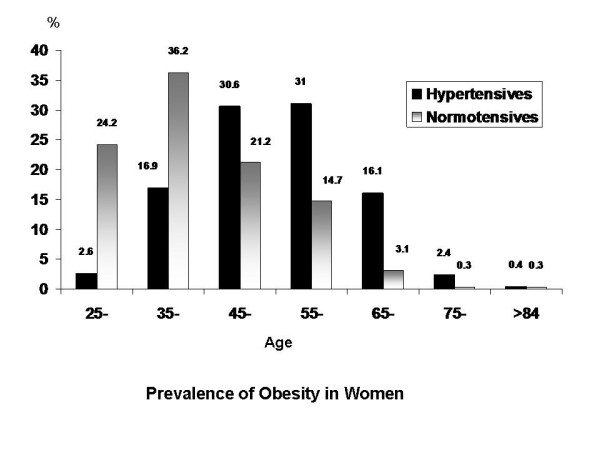
**Prevalence of Abdominal Obesity(%) in Different Age Decades in Normotensive and Hypertensive Women**.

Using the present WC cutoff points determined by the factor analysis approach, the prevalence of obesity in the study population was 50%. There was no difference in prevalence between men and women (48% and 51.5% respectively). The prevalence of abdominal obesity in NT men, HT men, NT women and HT women were 33.8%, 54.6%, 42.8% and 55.9% respectively. Figure (5 and 6) shows the prevalence of abdominal obesity in different age decades in NT and HT subjects. Above the age of 45 years, the prevalence of abdominal obesity was higher in HT compared to NTsubjects and reached the highest prevalence rate around middle age.

The prevalence rates of abdominal obesity differed according to the SES and urban/rural residence. Table 4 shows the prevalence of abdominal obesity in different SES groups and in urban/rural areas. Abdominal obesity was more prevalent in urban than rural areas and in middle and high SES compared to low SES.

**Table 4:** Prevalence of abdominal obesity by the new cutoff points in different socioeconomic classes and urban/rural areas among NT and HT men and women

	Low SES N (%)	Mid SES N (%)	High SES N (%)	Urban N (%)	Rural N (%)
NT and HT males	121 (32.7)	271 (55.1)	71 (69.6)	393 (56.3)	70 (26.3)
NT females	62 (31.2)	103 (52.3)	20 (55.6)	153 (50.8)	32 (24.4)
HT females	163 (41.5)	270 (68.2)	52 (65.8)	419 (66.4)	66 (27.8)

NT and HT males were grouped together because they had similar WC cutoff point for abdominal obesity.

## Discussion

The diagnosis of abdominal obesity is based on measurement of WC, exceeding a certain threshold establishes the diagnosis. The available cutoff points were derived from the white populations. These cutoff points may not be applicable for other countries because of variations in body proportions and body fat distribution in different ethnic groups. Reported WC thresholds for abdominal obesity were population and gender- specific (6). The IDF recommended that the cutoff point used for the WC to define abdominal obesity should be different among different ethnic groups (3,6). The IDF and WHO recommend the use of the European cutoff points (94 cm for men and 80 cm for women) in Egypt and other Eastern Mediterranean countries until more specific data are available (3,6).

In the present study, we tried to report specific WC cutoff points associated with increased cardiometabolic risk among a Middle Eastern population. We used these cutoff points to provide information about the prevalence of abdominal obesity in a sample of Egyptians representative of all socioeconomic strata and geographic regions and including both NT and HT men and women.

To determine the WC cutoff point, we used the factor analysis approach. This approach was used by Razak et al (8) to define obesity cutoff points in multiethnic population, where ethnic-specific BMI cutoff points were derived through the use of key cardiometabolic RFs as outcomes. It simultaneously incorporates the varying markers used to measure a RF, is less dependent on the population distribution of the variables, and can be used to characterize continuous RFs (7). We concluded that the WC cutoff points should be 93.5 cm for NT and HT men and 91.5 cm and 92.5 cm for NT and HT women respectively. The cutoff point for men is almost similar to the Europeans (94 cm) but much less than the American cutoff point (102 cm). In women, there is a big difference between our cutoff points (91.5 cm and 92.5 cm for NT and HT respectively) and that of the Europeans (80 cm) but is close to that of the Americans (88 cm) (3,6). Our study shows a small difference between the cutoff points for men and women (1-2 cm) in contrast to a larger difference in the European and American cutoff points (14 cm in both populations) (3,6). A small gender difference in cutoff points was also found in the Korean population (13), while the cut off points were greater in Japanese women (90 cm), compared to men (85 cm) (14).

Abdominal obesity is a common health problem in Egypt, with a prevalence rate of 33.8% and 54.6% for NT and HT men respectively, and 42.8% and 55.9% for NT and HT women respectively, when applying our new WC cutoff points derived from the present study. These prevalence rates are higher than that reported for US men (WC > 102 cm, 29.8%) but close to that of women (WC > 88 cm, 46.3%) (15). The cutoff point proposed by IDF and WHO to define abdominal obesity in women is lower than those found in our study (3,6). Therefore, prevalence rates of abdominal obesity in Egyptian women based upon the new cutoff points are different from those based on the IDF guidelines. The Egyptian prevalence rates exceeded those reported in many European and African countries (16, 17). In European men and women, abdominal obesity defined according to cutoff values between 90-102 cm for men and 80-92 cm for women, was 23 and 65% in Spain (17), 8 and 13% in France (18), 21 and 24% in Belgium (19), and 18 and 39% in Turkey (20). In Cameroon, the prevalence of abdominal obesity was 18% in men (WC > 94 cm) and 66% in women (WC > 80 cm) (21).

The association between increase in WC and cardiometabolic RFs, though reported in developed countries, was rarely addressed in developing countries with different genetic, lifestyle and environmental backgrounds. The present study is the first to report specific abdominal cutoff points estimated with waist girth and are associated with increased cardiovascular risk among Egyptians. It is also the first to provide information about the prevalence of abdominal obesity in nationwide sample of Egyptians representative of all socioeconomic strata and geographic regions and including NT and HT individuals.

We investigated the correlation between WC and cardiometabolic RFs such as type 2 diabetes mellitus, increased LDL-C and TG, decreased HDL-C and increased LVMI determined echocardiographically. Our data shows a continuous increase in RFs levels with higher WC in a linear fashion. However, this linear trend was not significant for all RFs and there was a gender difference. On the other hand, prevalence of abdominal obesity was higher in HT than NT subjects above the age of 45 years.

The higher prevalence of obesity in urban area and in upper socioeconomic class is possibly related to the rich caloric diet and more sedentary lifestyle in this population compared to rural residents and those in the lower socioeconomic group.

Given the continuous increase in prevalence of cardiovascular RFs with increasing WC shown by the present data, it must be acknowledged that all the cutoff points are arbitrary; no threshold in WC can be determined below which there is no increase in CVD risk and above which there is a uniform increase in risk.

WC was a stronger predictor of diabetes than BMI, and could identify persons who are at a greater cardiometabolic risk more than BMI (22, 23). Abdominal obesity was reported to be associated with greater risk of hypertension, diabetes and coronary artery disease (24-27). The additional health risk explained by WC likely reflects its ability to act as a surrogate for abdominal- and in particular- visceral fat (28).

CVD is now the main cause of mortality among Egyptians, being responsible for 47% of deaths according to the Egyptian Central Agency for Mobilization and Statistics (29). The age standardized mortality rates for CVD in Egypt were 560 per 100.000 populations in the year 2008 exceeding many European (France 118, Germany 211, Finland 401) and African countries (Ethiopia 435, Djibouti 533) according to WHO statistics 2008 (30). Ischemic heart disease being responsible for 21% of all deaths in Egypt in the year 2002 (WHO statistics: Death and DALY estimates by cause, 2002) (31). The high prevalence rates of abdominal obesity and associated cardiometabolic RFs among Egyptians is possibly an important factor behind the high CV risk.

Our study has a number of limitations. The cross-sectional nature of the study precludes definite causal inference about the association between the WC and RFs. The data are limited to the Egyptian population and may not represent the WC cutoff values required for definition of abdominal obesity in other Middle Eastern countries. Additional work in other populations is required to determine the generalizability of these thresholds. We used ROC analysis to define the optimal cutoff values of the WC associated with RFs, however, the discriminatory power was relatively weak. The sensitivity and specificity using these cutoff values were rather modest. Setting a cutoff level to obtain a high sensitivity of at least 80% will considerably decrease specificity. The cutoffs recommended here were obtained at WC values that best balanced sensitivity and specificity. The areas under the curves (AUC) ranged between 0.63 and 0.78, which reflects fair to good classification boundaries, the *P *value was highly significant (*P *< 0.001) in all groups and with narrow confidence intervals.

The present study did not have direct measures of body fatness or fat distribution. Because WC is considered a surrogate for body fatness and fat distribution, future research is needed into racial-ethnic differences in the relation between WC and actual body fatness and body fat distribution. Such studies would assist in determining whether some populations preferentially deposit abdominal fat and would help to develop WC cutoff points.

The study by the Emerging Risk Factors Collaboration (32) concluded that BMI, WC and waist-to-hip ratio, whether assessed singly or in combination, do not importantly improve CVD risk prediction in people in developed countries when additional information is available for systolic blood pressure, history of diabetes and lipids. The study does not underestimate the value of our work, since the definition of WC cutoff points and diagnosis of abdominal obesity is required, not only for risk stratification and prediction of future CV events, but also as essential component of the metabolic syndrome (3). Being an increasingly important condition and one established CVD risk factor, its prevalence is not known in many countries. The identification of appropriate ethnic-specific targets, whether BMI or WC, will have a major impact on the prevalence of metabolic syndrome and cardiometabolic disease risk at a population level. Furthermore, one should not ignore the need for healthy lifestyle to control abdominal obesity in the community. The target WC associated with increased CV risk and normal levels should be defined for each population. Guidelines should be population-specific regarding the optimal WC which is recommended in public education programs. There is the practical consideration of what threshold justifies the expenditure of national medical resources for clinical intervention (e.g. nutritional and physical activity counseling) in contrast to public health interventions (6).

## Conclusion

This is the first report of specific abdominal obesity cutoff points in a Middle Eastern country. There is a high prevalence rate of abdominal obesity among Egyptians which is associated with increased prevalence of cardiometabolic RFs.

Long-term prospective studies are required to reach more reliable WC cutoff points for different ethnic groups. Cross-sectional and longitudinal data relating WC to the risk of both CVD and type-2 diabetes are needed. Epidemiologic studies are also needed to understand the causes of CVD internationally and determine its magnitude in low-income countries. The data in the Egyptian study might be useful in this regard. Also, there is an urgent need in Egypt to describe a public health strategy to adequately enhance behavioral transformation necessary for total and abdominal obesity reduction.

## Competing interests

The authors declare that they have no competing interests.

## Authors' contributions

MMI is the principle investigator and has formulated the study design, reviewed literature, designed data collection and tabulation and wrote the manuscript, AA: participated in the data tabulation and statistical analysis, HG shared in the data analysis and statistical work, MAN contributed to the data collection and data entry.

All authors have read and approved the final manuscript.

## Pre-publication history

The pre-publication history for this paper can be accessed here:

http://www.biomedcentral.com/1471-2261/11/53/prepub
